# First evidence of polyandry for amazonian stingray (*Potamotrygon wallacei* Carvalho, Rosa & Araújo 2016) based on genome-wide SNP data

**DOI:** 10.1007/s11033-026-11695-0

**Published:** 2026-03-20

**Authors:** Larissa Emily Santos da Silva, Pedro Senna Taylor Bittencourt, Fábio de Lima Muniz, Wallice Luiz Paxiúba Duncan, Tomas Hrbek, Izeni Pires Farias

**Affiliations:** 1 Laboratório de Evolução e Genética Animal (LEGAL), Departamento de Genética, Universidade Federal do Amazonas (UFAM), Manaus, Amazonas Brazil; 2 Divisão Técnico-Ambiental (Ditec), Instituto Brasileiro do Meio Ambiente e dos Recursos Naturais Renováveis (IBAMA), Roraima, Brazil; 3 Instituto de Ciências Exatas e Naturais (ICEN), Universidade Federal de Rondonópolis (UFR), Mato Grosso, Brazil; 4 Laboratório de Morfologia Funcional, Universidade Federal do Amazonas (UFAM), Manaus, Amazonas Brazil; 5 Department of Biology, Trinity University, San Antonio, TX USA

**Keywords:** Arraia cururu, SNPs, Genetic diversity, Multiple paternity

## Abstract

**Background:**

The Amazonian freshwater stingray *Potamotrygon wallacei*, endemic to the Rio Negro basin, although heavily exploited in ornamental fish trade, is largely neglected in terms of basic biology, including its reproductive system. Polyandry is a widespread reproductive strategy in elasmobranchs, however, evidence from freshwater potamotrygonids is scarce. To address this knowledge gap, the present study developed highly polymorphic single nucleotide polymorphism (SNP) genomic markers for *P. wallacei* and applied them to investigate kinship patterns in wild-caught gravid females and their litters.

**Methods and Results:**

This study used genomic polymorphic SNP markers in 18 individuals from five family groups to assess kinship patterns. The average gene diversity was low, however, He (0.435–0.476) and Ho (0.530–0.653) were higher, with Ho > He, suggesting small-sample bias or heterozygote excess from family-level sampling. Polyandry was detected in three families, while two families exhibited monogamy.

**Conclusions:**

This molecular approach allowed the discovery of polyandry in *P. wallacei*, providing novel insights into its reproductive biology and contributing to broader understanding of mating system evolution in Neotropical freshwater elasmobranchs.

## Introduction

*Potamotrygon wallacei* Carvalho, Rosa & Araújo 2016, known as the “cururu stingray”, is an endemic species native to the Rio Negro basin in the Amazon. It is the smallest freshwater stingray of the genus *Potamotrygon*, which makes it popular in the aquarium hobby. This species prefers shallow, acidic blackwaters (pH < 5) of flooded forests (igapós) and streams (igarapés) with sandy bottoms covered in leaf litter and other organic matter. Limited data indicate that there are no significant ontogenetic shifts in niche utilization between juveniles and adults [1]. The species is extensively exploited for the international aquarium market, yet its conservation status remains poorly understood, with no current assessment on the International Union for Conservation of Nature (IUCN) Red List, raising concerns about potential overexploitation and population declines.

Elasmobranchs exhibit diverse mating systems, but molecular studies over the past two decades have revealed that female polyandry is a widespread reproductive strategy and likely the predominant strategy across the group [2]. This pattern has been documented in marine species [3], as well as in some freshwater potamotrygonids [4].

Despite these insights from other elasmobranchs, the mating system of freshwater stingrays of the genus *Potamotrygon* remains largely unstudied, representing a significant gap in our understanding of their reproductive biology. To address this, in the present study we developed single nucleotide polymorphism (SNP) markers for the cururu stingray, *Potamotrygon wallacei*, and applied them to assess kinship patterns in litters of wild-caught females. This approach provides the first molecular evidence of polyandry in this species and contributes to broader knowledge of reproductive strategies in Neotropical freshwater elasmobranchs.

## Materials and methods

All animal procedures were conducted in accordance with the ARRIVE guidelines (Percie du Sert et al., 2020) and Brazilian national legislation (CONCEA – Conselho Nacional de Controle de Experimentação Animal, Lei Arouca nº 11.794/2008). Eighteen samples of *P. wallacei* were collected in Lagoa de Cuba (0°56’24.10"S, 62°55’59.14"W) using fishing gear and euthanized with a 0.5% benzocaine solution. The study was authorized by the Chico Mendes Institute for Biodiversity Conservation (ICMBio) license No. 32360-1. All animal handling protocols were approved by the Ethics Committee on Animal Experimentation at the Federal University of Amazonas (protocol No. 007/2019) following CFBio Resolution 301 (December 8, 2012). Only litters with at least three pups were used in the analyses. Five family groups were analyzed: CA12 (*N* = 3), CA26 (*N* = 4), CA47 (*N* = 4), CA51 (*N* = 4) and CANI (*N* = 3, which did not include the mother). Muscle tissue was used to extract genomic DNA using the CTAB protocol [5]. The genomic library involved digesting the DNA with the restriction enzymes *SdaI* and *Csp6*, then ligated to Illumina compatible adapters. The ligated DNA fragments were indexed with Q7 and Q5 indices via PCR, and then the fragment size range of 400–630 bp was selected using a Pippin-Prep (Sage Science) prior to sequencing on Illumina NovaSeq X.

The SNPs were obtained by processing the raw data in FASTQ format using DiscoSnpRad [6]. Quality-based sequence filtering and conversion of the VCF file to other formats was performed using the vcf2others.R package (https://github.com/legalLab/vcf2others) of R (R Core Team, version v4.5.1). We filtered the raw VCF file: (1) Excluding individuals with over 90% missing data; (2) Removing SNPs with rare alleles below 3% frequency; (3) Eliminating SNPs with less than 10x coverage; (4) Retaining only unlinked SNPs; (5) Removing SNPs with more than 20% missing data; and (6) Excluding individuals with over 30% missing data. After quality filtering, the VCF file containing 1,664 SNPs was converted into the appropriate input formats for downstream analyses. The VCF analyzed in this study is available at https://github.com/legalLab/publications.

We used the Arlequin 3.5 program [7] to calculate genetic parameters, such as average genetic diversity (AGD), expected heterozygosity (He), and observed heterozygosity (Ho). To estimate pairwise kinship relationships within the family groups we utilized the R1 and KING-robust estimators implemented in the NGSRelate program [8], following the robust allele frequency-free inference framework developed by Waples et al. [9]. Specifically, R1 quantifies the proportion of sites where two individuals share exactly one identical-by-state allele (IBS). The KING-robust kinship coefficient provides a complementary measure of overall relatedness resilient to SNP ascertainment bias and low sequencing depth. The expected ranges for kinship categories (full-sibling/parent-offspring (FS/PO): R1 ≈ 0.5–1.0, KING ≈ 0.25–0.5; half-sibling (HS): R1 ≈ 0.2–0.5, KING ≈ 0.08–0.15; first-cousin (C1): R1 ≈ 0.1–0.35, KING ≈ 0.04–0.08) used in the *P. wallacei* kinship analysis were derived based on the general principles of the allele frequency-free inference framework described in Waples et al. [9]. The results were visualized by plotting R1 against KING-robust values for each pairwise comparison in R v4.5.1 [10].

## Results and discussion

The low average gene diversity (AGD) values obtained in this study for *P. wallacei* (ranging from 0.231 to 0.277 across families; Table 1) could be attributed to the small sample size (five families with 3–4 offspring each, totaling 19 individuals), which may limit the capture of allelic variation. The results are presented in Table 1. Although the low genetic diversity observed in our limited family sample cannot be directly attributed to ornamental fishing, this explanation remains plausible given the intense harvest of the species, its restricted endemic range, and low fecundity, all of which increase susceptibility to bottlenecks in exploited elasmobranchs [11]. Broader population-genetic sampling is needed to test this hypothesis. On the other hand, He (0.435–0.476) and Ho (0.530–0.653) are higher than AGD, with Ho exceeding He—a pattern potentially indicative of small-sample bias or heterozygote excess due to family-level sampling. These heterozygosity levels are higher than those reported by Belém [12], who documented average Ho = 0.39 and He = 0.38 in *P. wallacei* individuals from five populations (total of 25 individuals) in the Rio Negro basin using 2,089 SNPs. Similarly, the heterozygosity levels for *P. wallacei* in this study exceed those observed for the marine species *Pseudobatos percellens* (Ho = 0.395, He = 0.342), based on SNPs [13], possibly due to methodological differences in SNP discovery and filtering. Elasmobranchs generally exhibit low genetic diversity owing to their slow molecular evolution rates and K-strategist life history traits, including low fecundity, long gestation periods, and extended generation times [14].

**Table 1:** Values of R1 and KING-robust statistics, kinship categorization, and mating system for potamotrygon wallacei family groups.

Family	Number of SNPs	AGD	He	Ho	Pair	R1	KING-Robust	Kinship category	Mating system
CA12 (*N* = 3)	845	0.255 (± 0.145)	0.476 (± 0.116)	0.595 (± 0.274)	0–1	0.974	0.318	FS	Monogamy
					0–**2**	0.549	0.216	PO	
					1–**2**	0.512	0.157	PO	
CA26 (*N* = 4)	706	0.277 (± 0.149)	0.441 (± 0.123)	0.530 (± 0.275)	**3**–4	0.442	0.134	PO	Polyandry
					**3**–5	0.373	0.127	PO	
					**3**–6	0.385	0.141	PO	
					4–5	0.425	0.143	HS	
					4–6	0.700	0.232	FS	
					5–6	0.553	0.241	FS	
CA47 (*N* = 4)	788	0.231 (± 0.125)	0.435 (± 0.126)	0.541 (± 0.274)	7–**8**	0.615	0.239	PO	Polyandry
					7–9	0.619	0.157	FS	
					7–10	0.808	0.267	FS	
					**8**–9	0.579	0.196	PO	
					**8**–10	0.322	0.137	PO	
					9–10	0.349	0.041	HS/C1	
CA51 (*N* = 4)	829	0.236 (± 0.127)	0.463 (± 0.121)	0.653 (± 0.297)	15–16	1.534	0.340	FS	Monogamy
					15–17	1.427	0.361	FS	
					15–**18**	1.149	0.348	PO	
					16–17	1.060	0.340	FS	
					16–**18**	1.261	0.358	PO	
					17–**18**	1.366	0.358	PO	
CANI (*N* = 3)	813	0.239 (± 0.135)	0.470 (± 0.115)	0.586 (± 0.270)	19–20	0.525	0.219	FS	Polyandry
					19–21	0.475	0.081	HS	
					20–21	0.599	0.238	FS	

Note: N= sample size; average gene diversity (AGD), expected (He) and observed (Ho) heterozygosity. Standard deviation in parentheses. PO= parent-offspring, FS= full-sibling, HS= half-sibling, C1 = first-cousin. Mothers are highlighted in bold

**Fig. 1:**
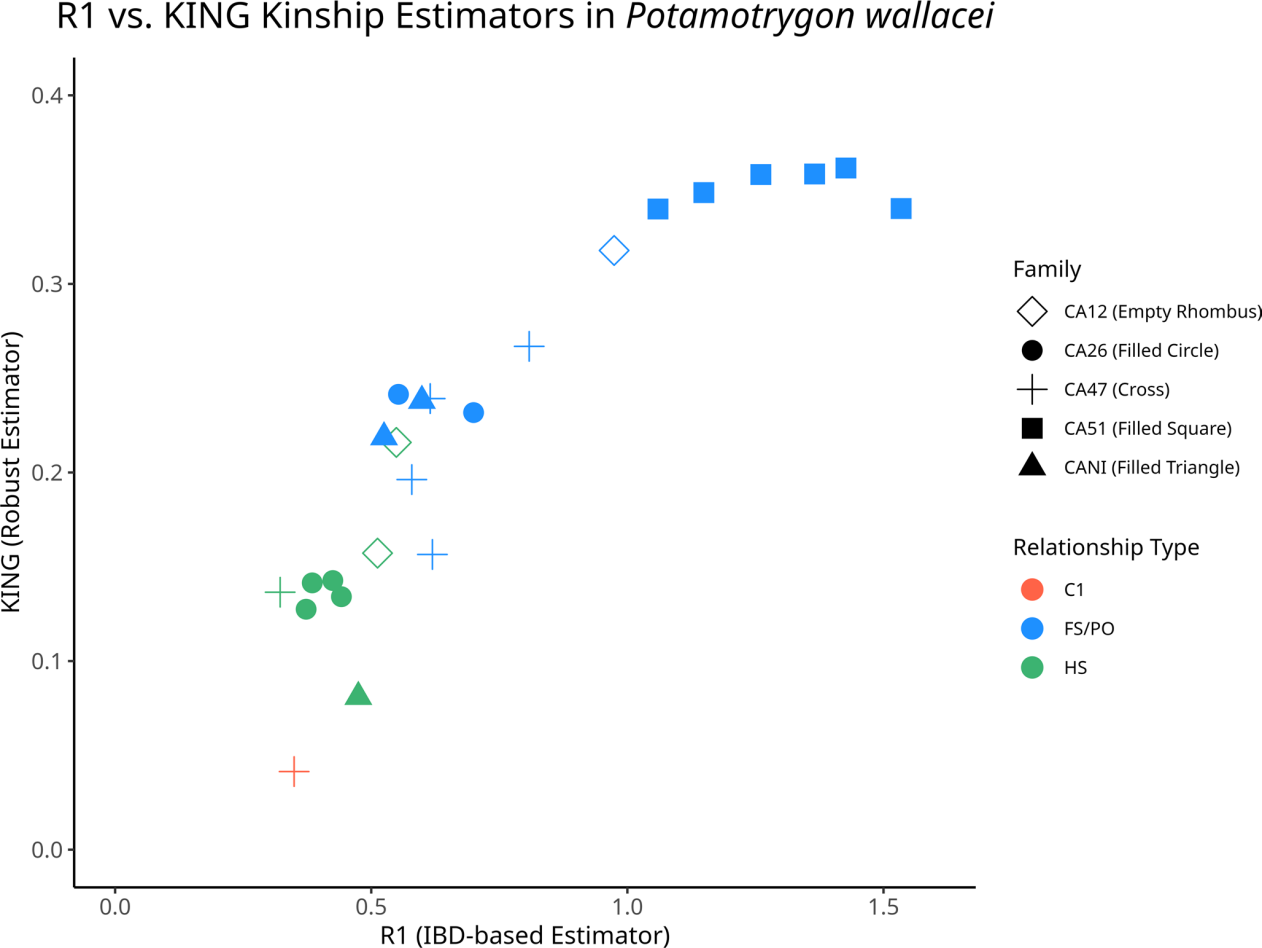
Relationship patterns among the five *Potamotrygon wallacei* family groups, estimated from genomic SNP data using the R1 and KING-robust estimators (Waples et al. 2019). Detected relationship categories include parent–offspring (PO), full-sibling (FS), half-sibling (HS), and first-cousin (C1) pairs

By plotting R1 against KING-robust values for each pairwise comparison (Fig. 1), the kinship analysis identified 24 pairwise relationships distributed among five families, based on 706–845 SNPs per group, revealing distinct clusters: full-sibling/parent-offspring (FS/PO) pairs exhibited higher R1 (typically 0.5–1.5) and KING (0.2–0.36) values, half-sibling (HS) pairs showed intermediate values (R1 ~ 0.3–0.55, KING ~ 0.08–0.24), and first-cousin (C1) pairs had the lowest KING (~ 0.04). Polyandry was detected in families CA26, CA47, and CANI through the presence of HS pairs, while CA12 and CA51 displayed monogamy with anomalously high R1 values (> 1) for the latter, suggestive of inbreeding (see Table 1).

Although the maternal sample was unavailable for the CANI group, categorizations rely on R1 (0.475) and KING ranges, with HS (sibling pair 19–21) indicating polyandry. The family group CA51 exhibited parent-offspring (PO) relationships with the mother, and full-sibling (FS) relationships among siblings, suggestive of monogamous mating.

For elasmobranchs, a low frequency of genetic monogamy is expected because species in this group do not form stable pairs after copulation and do not provide postnatal parental care to their offspring [15]. Multiple paternity in families CA26, CA47, and CANI, evidenced by half-sibling (HS) pairs, suggests that polyandry may be a prevalent mating strategy in *P. wallacei*. This strategy likely enhances genetic diversity through mixed-paternity litters facilitated by ovoviviparous reproduction. Polyandry can be explained by the following hypotheses: the genetic benefits of increased offspring diversity [16] or convenience polyandry, where females mate with multiple partners due to differing mating rates between the sexes [17]. Polyandry may also arise from coercive mating, a phenomenon that seems to be prevalent among elasmobranchs [18].

Polyandrous behavior may be facilitated by male competition and sperm storage, both of which are well documented in elasmobranchs [2, 3]. Shibuya et al. [19] reported that ovulating females of *Potamotrygon motoro* release cloacal pheromones that signal reproductive status and attract males, likely promoting copulation. This pheromonal communication may increase the number of males mating with a single female, favoring polyandry. Given the close relationship between *P. motoro* and *P. wallacei*, similar mechanisms may operate in *P. wallacei*, potentially explaining the observed genetic evidence of polyandry and offering broader insight into potamotrygonid reproductive ecology.

Genetic monogamy and polyandry have evolved independently multiple times within Elasmobranchii [20]. These two extremes may serve as alternative adaptive strategies favored by distinct ecological and biological conditions [18]. It is well known that multiple paternity may enhance reproductive success by increasing effective population size (Ne), preserving genetic diversity, and producing more viable offspring per litter [16]. However, in elasmobranchs, the consequences of polyandry for reproductive success are, in general, context-dependent: mutually beneficial polyandry can increase offspring genetic diversity and litter viability, whereas coercive polyandry may reduce female fitness through injury, stress, or mating with suboptimal males. Even in consensual cases, polyandry presents the risk of disrupting co-adapted genetic complexes [18].

## Conclusions

This study provides the first molecular evidence of multiple paternity in *Potamotrygon wallacei*, indicating that polyandry is a biologically relevant component of its reproductive strategy. The occurrence of both polyandrous and monogamous families suggests true intraspecific variation in mating patterns, likely influenced by ecological or demographic factors rather than sample size alone. Future studies integrating behavioral observations with larger-scale genetic and ecological analyses are needed to identify the drivers of polyandry and to inform effective conservation strategies for this exploited freshwater stingray.

## Data Availability

The VCF data analyzed in this study is available at https://github.com/legalLab/publications.

## References

[1] Carvalho MRD, Rosa RS, Araújo MLGD (2016) A new species of Neotropical freshwater stingray (Chondrichthyes: Potamotrygonidae) from the Rio Negro, Amazonas, Brazil: the smallest species of *Potamotrygon*. Zootaxa 4107. 10.11646/zootaxa.4107.4.5 10.11646/zootaxa.4107.4.5 27394840

[2] Fitzpatrick JL, Kempster RM, Daly-Engel TS, Collin SP, Evans JP (2012) Assessing the potential for post‐copulatory sexual selection in elasmobranchs. J Fish Biol 80:1141–1158. 10.1111/j.1095-8649.2012.03256.x 22497376 10.1111/j.1095-8649.2012.03256.x PMC3842027

[3] Lyons K, Chabot CL, Mull CG, Paterson Holder CN, Lowe CG (2017) Who’s My Daddy? Considerations for the influence of sexual selection on multiple paternity in elasmobranch mating systems. Ecol Evol 7:5603–5612. 10.1002/ece3.3086 28808540 10.1002/ece3.3086 PMC5551082

[4] Torres Y, Charvet P, Faria VV, De Castro ALF (2022) Evidence of multiple paternity for the endemic Xingu River stingray. J Fish Biol 100:1315–1318. 10.1111/jfb.15038 35292972 10.1111/jfb.15038

[5] Doyle JA, Doyle J A rapid DNA isolation procedure for small quantities of fresh leaf tissue. Phytochem. Bull. 19: 11–15

[6] Gauthier J, Mouden C, Suchan T, Alvarez N, Arrigo N, Riou C et al (2020) DiscoSnp-RAD: de novo detection of small variants for RAD-Seq population genomics. PeerJ 8:e9291. 10.7717/peerj.9291 32566401 10.7717/peerj.9291 PMC7293188

[7] Excoffier L, Lischer HEL (2010) Arlequin suite ver 3.5: A new series of programs to perform population genetics analyses under Linux and Windows. Mol Ecol Resour 10:564–567. 10.1111/j.1755-0998.2010.02847.x 21565059 10.1111/j.1755-0998.2010.02847.x

[8] Korneliussen TS, Moltke I (2015) NgsRelate: a software tool for estimating pairwise relatedness from next-generation sequencing data. Bioinformatics 31:4009–4011. 10.1093/bioinformatics/btv509 26323718 10.1093/bioinformatics/btv509 PMC4673978

[9] Waples RK, Albrechtsen A, Moltke I (2019) Allele frequency-free inference of close familial relationships from genotypes or low‐depth sequencing data. Mol Ecol 28:35–48. 10.1111/mec.14954 30462358 10.1111/mec.14954 PMC6850436

[10] R Core Team. R: A language and environment for statistical computing. Vienna, Austria: R Foundation for Statistical Computing (2025) https://www.r-project.org

[11] Dulvy NK, Pacoureau N, Rigby CL, Pollom RA, Jabado RW, Ebert DA et al (2021) Overfishing drives over one-third of all sharks and rays toward a global extinction crisis. Curr Biol 31:4773–4787e8. 10.1016/j.cub.2021.08.062 34492229 10.1016/j.cub.2021.08.062

[12] Belém R (2016) Diversidade morfológica e genética de arraia cururu (*Potamotrygon wallacei* Carvalho, Rosa e Araújo, uma espécie de igarapé. Master Thesis, Universidade Federal do Amazonas. 2020. https://tede.ufam.edu.br/handle/tede/8074

[13] de Souza B (2021) C. Composição genética da raia viola *Pseudobatos percellens* (Elasmobranchii: Rhinobatidae) utilizando SNPs. Master Thesis, Universidade Estadual Paulista (Unesp). http://hdl.handle.net/11449/215846

[14] Sendell-Price AT, Tulenko FJ, Pettersson M, Kang D, Montandon M, Winkler S et al (2023) Low mutation rate in epaulette sharks is consistent with a slow rate of evolution in sharks. Nat Commun 14:6628. 10.1038/s41467-023-42238-x 37857613 10.1038/s41467-023-42238-x PMC10587355

[15] Pratt HL, Carrier JC (2001) A review of elasmobranch reproductive behavior with a case study on the Nurse Shark, *Ginglymostoma cirratum*. Environ Biol Fishes 60:157–188. 10.1023/A:1007656126281

[16] Jennions MD, Petrie M (2000) Why do females mate multiply? A review of the genetic benefits. Biol Rev Camb Philos Soc 75:21–64. 10.1111/j.1469-185X.1999.tb00040.x 10740892 10.1017/s0006323199005423

[17] Fromonteil S, Marie-Orleach L, Winkler L, Janicke T (2023) Sexual selection in females and the evolution of polyandry. Patricelli GL, editor. PLOS Biol. ;21: e3001916. 10.1371/journal.pbio.3001916 10.1371/journal.pbio.3001916 PMC9831318 36626380

[18] Gayford JH, Soares KD, Berio F (2025) Sexual ornamentation and weapons of sexual conflict in cartilaginous fishes. Rev Fish Biol Fish 35:2217–2233. 10.1007/s11160-025-10000-9

[19] Shibuya A, Duncan WP (2022) Pre-copulatory bite wounds as evidence of aggressive competition for mating in the Neotropical freshwater stingray *Potamotrygon motoro*. Acta Amaz 52:45–48. 10.1590/1809-4392202101762

[20] Gayford JH, Flores-Flores EM (2024) No evidence for population-level benefits of polyandry in sharks and rays. PLoS ONE 19:e0308141. 10.1371/journal.pone.0308141 39231154 10.1371/journal.pone.0308141 PMC11373851

